# Ventilation and Warming

**Published:** 1873-05

**Authors:** 


Ventilation and Warming.—In a lecture on ventilation, lately delivered before the Franklin Institute, Mr. L. W. Leeds, after detailing the abominations he encountered in his examination of the ventilating arrangements of the Treasury building at Washington, gives the following practical directions concerning proper provisions for ventilation and warming in the construction of buildings: First, never have long underground fresh-air ducts. Second, never allow a sewer, soil-pipe, foul-air flue, or smoke flue, to come near the fresh-air-supply-flue, for fear of some connection being made between them by carelessness or accident. Never heat a building exclusively by currents of warm air. Fourth, always put the heating-flues on the outside walls instead of the inside walls. Fifth, endeavor strenuously to avoid the fresh-air chamber becoming a receptacle for all the rubbish of a filthy cellar.—*Medical Times.*

Charleston Medical Journal and Review.—We have received the first number of the above journal, and welcome it to our exchange list, and extend to its editors, Drs. Porcher and Kinloch, our earnest wishes for its successful establishment. We can, without hesitation, from our personal knowledge of its conductors, assure the profession that it will be well deserving their patronage.

